# Advanced Nursing Practice for Older People

**DOI:** 10.1155/2013/860167

**Published:** 2013-07-29

**Authors:** Kaja Põlluste, Pirkko Routasalo, Lisbeth Fagerström, Lis Wagner

**Affiliations:** ^1^Department of Internal Medicine, University of Tartu, L. Puusepa 6, 51014 Tartu, Estonia; ^2^Department of General Practice and Primary Health Care, University of Helsinki, P.O. Box 33 (Yliopistonkatu 4), 00014 Helsinki, Finland; ^3^Faculty of Health Sciences, Buskerud University College, Papirbredden, Grønland 58, 3045 Drammen, Norway; ^4^Research Unit of Nursing, Institute of Clinical Research, University of Southern Denmark, Campusvej 55, 5230 Odense M, Denmark

The world population is rapidly aging. Between 2000 and 2050, the proportion of the world's population over 60 years will double from about 11% to 22%. The absolute number of people aged 60 years and over is expected to increase from 605 million to 2 billion over the same period [1]. Although more developed countries have the oldest population profiles, the vast majority of older people—and the most rapidly aging populations—are in less developed countries. Between 2010 and 2050, the number of older people in less developed countries is projected to increase more than 250 percent, compared with a 71-percent increase in the developed countries [2].

Older people are considered one particular group of the population which needs and uses many health services of different nature, and the use of services is increasing with age. The aging of population has also resulted in increased interest in long-term care—in home care as well as in institutional care (residential and community care). The rising demand for long term-care is latest described in a three-year EU project titled INTERLINKS—health systems and long-term care for older people involving 13 countries in modelling INTERfaces and LINKS between prevention and rehabilitation to indicate new approaches and to improve policy and practice in long-term care [3]. To be responsive to the needs of older people, the continuity and quality of care should be improved by more effective management of care and improve the communication as well as interaction between patients, their family members, care providers, and politicians. It is important that the health services provided to the older population be responsive not only to their physical but also to the mental and emotional needs. In this process the nurses have a remarkable role, which, however, sometimes may not to be noted enough.

The papers published in this issue cover different fields of the organisation and content of nursing for older people from different parts of the world. 

The provision of care for older people has undergone several changes and reforms to find best solutions and balance between existing resources and increasing demands. For example, S. Kato et al. propose models for designing long-term care service plans and care programs for older people in Japan; in the country, where more than 60% of population is aged over 60. One of the most important aspects for older persons is the independency of decision making for their future care and maintaining the autonomy, regardless of staying home with help from the municipality or moving to the nursing home. These aspects are discussed in three papers (A. Breitholtz et al., M. Riedl et al., and D. Goodridge). Also, some specific areas are presented as, for example, oral health care from the nursing perspective (K. Salamone et al.) and coping with the partner's health problems (S. Marnocha and M. Marnocha). Finally, besides those who receive the care, one may not forget the caregivers too. In this issue, two research papers from Scandinavia focus on the nurses and caregivers experiences and ethical values (M. Frilund et al. and S. Salin et al.).

We hope that this issue provides interest to broad audience not only for nursing professionals, but also for other health and social care professionals while advanced nursing for older people like any other activity in health care can be most successful in team work.

*Kaja Põlluste* *Kaja Põlluste* *Pirkko Routasalo* *Pirkko Routasalo* *Lisbeth Fagerström* *Lisbeth Fagerström* *Lis Wagner* *Lis Wagner*
